# Efficacy of single antibiotic therapy versus antibiotic combination in implant-free staphylococcal post-surgical spinal infections: a retrospective observational study

**DOI:** 10.1186/s12879-024-08977-y

**Published:** 2024-01-08

**Authors:** Amélie Lombès, Marie-Paule Fernandez-Gerlinger, Marc Khalifé, Najiby Kassis-Chikhani, Amira Jomli, Jean-Luc Mainardi, David Lebeaux, Marie Dubert

**Affiliations:** 1 Mobile infectious disease unit, Service de Microbiologie, AP-HP, Hôpital Européen Georges Pompidou, APHP-Centre, 20 rue Leblanc, 75015 Paris, France; 2 Orthopedic surgery unit, AP-HP, Hôpital Européen Georges Pompidou, APHP-Centre, 20 rue Leblanc, 75015 Paris, France; 3 Infection control unit, AP-HP, Hôpital Européen Georges Pompidou, APHP-Centre, 20 rue Leblanc, 75015 Paris, France; 4 Microbiology laboratory, AP-HP, Hôpital Européen Georges Pompidou, APHP- Centre, 20 rue Leblanc, 75015 Paris, France; 5 Paris Cité university, 75006 Paris, France; 6 3-FHU PROTHEE, Paris, France

**Keywords:** Staphylococcus, Spinal, Surgical site infections (SSIs), Implant-free, Single antibiotic, Combination antibiotic

## Abstract

**Background:**

Post-surgical spinal infections (pSSIs) are a serious complication of spinal surgeries, with *Staphylococcus* spp. being one of the most prominent bacteria identified. Optimal antimicrobial therapy for staphylococcal spinal infections without spinal implants is not well documented.

**Methods:**

This single center retrospective 7-year observational study described and compared the outcome (treatment failure or mortality rate one year after diagnosis) of 20 patients with staphylococcal-implant-free pSSI treated with single or combination antibiotics.

**Results:**

Median duration of treatment was 40 days (IQR 38–42), with 6 days (IQR 5–7) on intravenous antibiotics and 34 days (IQR 30–36) on oral therapy. Four patients (20%) underwent new surgical debridement, all due to surgical failure, and 1 patient died within the first year without significant differences between both treatment group.

**Conclusion:**

This study raises the possibility of single antibiotic therapy for patients with implant-free post-surgical spinal infections due to *Staphylococcus* spp.

**Supplementary Information:**

The online version contains supplementary material available at 10.1186/s12879-024-08977-y.

## Introduction

Post-surgical spinal infections (pSSI) are a common complication of spinal surgery [1], associated with high morbidity rates due to functional sequelae [2] and mostly caused by *Staphylococcus aureus* [1, 3]. The optimal antibiotic therapy for staphylococcal spinal infections is not well documented, especially in the absence of spinal implantation. The relevance of a systematic dual antibiotic therapy and in particular the use of rifampin in combination, although recommended by some, is discussed by others [4–7]. Given this poor level of evidence, our hospital local guidelines evolved in 2018 and recommended the use of single antibiotic therapy for implant-free staphylococcal spinal infections. We aimed to describe and compare the clinical outcome of staphylococcal implant-free pSSIs in patients treated with a single or a combination antibiotic therapy.

## Materials and methods

We conducted a monocentric, retrospective observational study at Georges Pompidou European hospital. All adult (≥ 18 years old) patients having a staphylococcal pSSI between 1st of January 2014 and 1st of May 2021 were included. Staphylococcal pSSIs were defined by association of clinical and/or biological signs following spinal surgery, as previously described [3], associated with microbiological positivity of surgical site cultures (≥ 1/5 for *Staphylococcus aureus;* ≥ 3/5 for coagulase-negative staphylococci). Exclusion criteria were retention of spinal implants after revision surgery, infections documented with other bacterial genus than *Staphylococcus* spp., death during the first 14 days of treatment or less than 12-month follow-up.

All patients were treated according to standard procedures and managed by the local antibiotic stewardship team. Empiric antibiotic treatment included vancomycin with cefepim and gentamicin for maximum 2 days as previously described [3], followed by targeted antibiotic therapy according to microbiological identification. Revision surgery was performed as previously described [3]. Any antibiotic change was considered significant if duration lasted more than 14 days.

New surgical interventions were classified as: (i) Surgical failure in case of early revision prior to the end of antibiotic therapy with no micro-organisms identified in culture (ii) Superinfection in case of new surgical procedure with identification of microorganisms different than the one previously identified, (iii) Persistent infection in case of early revision in the course of antibiotic intake with identification of the same microorganism as the one previously identified (iv) Relapse in case of revision after the end of initial antibiotic treatment with identification of the same microorganism as the one initially identified.

Microbiological failure was defined as any surgical revision due to iii) or iv), the occurrence of bacteremia with the same microorganism and no other identified cause or a suspected infection relapse based on clinical, radiological and microbiological data.

The primary endpoint was a composite criterion including the rate of microbiological failure and all-cause mortality 1 year after the diagnosis of staphylococcal pSSI. Long-term follow-up was performed by gathering electronic healthcare data, medical records of any follow-up consultations when available and telephone follow-up in the absence of any recorded medical data.

All statistical analysis were performed with R 4.2.1 software. Continuous variables were given as median (interquartile range, IQR) and analyses were made with Fisher’s exact test. Categorical variables were given as numbers and percentages and analyzed with Mann Whitney’s test. All statistical comparisons and tests for significance assumed two tails with *p*-value set at < 0.05.

The local ethics committee approved the study (CER APHP number #00011928, RGPD registration number 20,220,216,145,039). Detailed information letter about the study was sent to all participants.

## Results

We screened 201 patients with pSSI and excluded 181 patients due to retention of spinal implants (178 patients), polymicrobial infection (2 patients) or insufficient follow-up (one patient). Twenty patients with *Staphylococcus* pSSI were included in total with no significant difference in baseline characteristics between the single and the combination therapy group (Table 1). The initial surgeries performed were laminectomies in all cases, mostly related to degenerative causes for 19 (95%) patients. 2 patients had spinal fusion with initial implantation because of spinal instability. All patients presented local signs of pSSI and 6 (30%) had fever (T°> 38 °C) with no signs of severity (sepsis or septic shock). *Staphylococcus aureus* was isolated in 80% of surgical samples, all methicillin-susceptible **(**Table 1**).**

**Table 1:** Patients characteristics, clinical presentation and microbiology

	Overall (*N* = 20)	Single therapy (*N* = 11)	Combination therapy (*N* = 9)	*P* value
**Patients characteristics**				
Men (n, %)	10 (50.0%)	6 (54.5%)	4 (44.4%)	1.00
Age (year), (median [IQR])	60.0 [47.5;69.2]	60.0 [52.0;74.0]	57.0 [46.0;67.0]	0.27
BMI (kg/m^2^), (median [IQR])	26.0 [23.2;27.7]	26.1 [23.3;29.3]	25.3 [23.0;26.4]	0.69
eGFR (ml/min/1.73m^2^) (median [IQR])	94.5 [78.5;111]	95.0 [71.5;114]	91.0 [84.0;109]	0.76
Charlson comorbidity index (with age) (median [IQR])	2.00 [0.75;4.25]	4.00 [0.50;6.50]	2.00 [1.00;2.00]	0.17
**Primary surgery characteristics**				
Number of previous spinal surgery (median [IQR])	1.00 [1.00;1.00]	1.00 [1.00;1.00]	1.00 [1.00;1.00]	0.67
Surgical indication (n, %)				
Degenerative spine disease	19 (95.0%)	10 (90.9%)	9 (100%)	1.00
Vertebral metastasis	1 (5.00%)	1 (9.09%)	0 (0.00%)	
Laminectomy (n, %)	20 (100%)	11 (100%)	9 (100%)	
Spinal implants (n, %)	2 (10.0%)	2 (18.2%)	0 (0.00%)	
Extent of surgery (number of vertebrae involved) (median [IQR])	3.00 [2.00;4.00]	4.00 [2.50;4.50]	2.00 [2.00;3.00]	0.05
Initial ASA score (median [IQR])	2.00 [1.00;3.00]	2.00 [1.50;3.00]	1.00 [1.00;2.00]	0.20
**Post-surgical spinal infection**				
Delay between first surgery and revision surgery (days) (median [IQR])	22.5 [17.8;29.8]	23.0 [19.5;32.0]	22.0 [17.0;24.0]	0.45
Local signs of infection (n, %)	20 (100%)	11 (100%)	9 (100%)	1.00
Fever (n, %)	6 (30.0%)	4 (36.4%)	2 (22.2%)	0.64
Site of involvement (n, %)				0.22
Cervical spine	4 (20%)	3 (27.3%)	1 (11.1%)	
Thoracic spine	1 (5%)	1 (9.09%)	0 (0%)	
Lumbar spine	14 (70%)	7 (63.6%)	7 (77.8%)	
Sacral spine	3 (15%)	0 (0%)	3 (33.3%)	
Number of vertebrae involved (median [IQR])	2.50 [2.00;4.00]	4.00 [2.00;4.00]	2.00 [2.00;3.00]	0.13
Blood cultures performed (n, %)	4 (20%)	3 (27%)	1 (11%)	0.59
Positive blood culture, (n, %)	0	0	0	
Positive surgical cultures if surgery performed (n, %)	20 (100%)	11 (100%)	9 (100%)	1.00
Implant removal (n, %)	2 (10.0%)	2 (18.2%)	0 (0.00%)	
***Staphylococcus aureus*** (n, %)	16 (80.0%)	9 (81.8%)	7 (77.8%)	1.00
Wild type* (n, %)	4 (25.0%)	3 (33.0%)	1 (14.0%)	
methicillin susceptible (n, %)	16 (100%)	9 (100%)	7 (100%)	
fluoroquinolone resistant (n, %)	0	0	0	
rifampicin resistant (n, %)	0	0	0	
**Coagulase negative staphylococci (%)**	4 (20.0%)	2 (18.2%)	2 (22.2%)	1.00
methicillin susceptible (n, %)	2 (50%)	1 (50%)	1 (50%)	
methicillin resistant (n, %)	2 (50%)	1 (50%)	1 (50%)	
fluoroquinolone resistant (n, %)	2 (50%)	1 (50%)	1 (50%)	
rifampicin resistant (n, %)	0	0	0	

*Wild-type Staphyloccus aureus were defined as strains susceptible to all antibiotics (penicillin G, cefoxitin, moxalactam, erythromycin, pristinamycin, clindamycin, rifampin, fusidic acid, tetracycline, kanamycin, tobramycin, gentamicin, fosfomycin, levofloxacin, linezolid, vancomycin, teicoplanin, trimethoprim-sulfamethoxazole, fosfomycin, nitrofuran, novobiocin) tested by disc diffusion method on Mueller-Hinton agar.

Abbreviations: eGFR, estimated Glomerular filtration rate; BMI, body mass index; IQR, interquartile range. Continuous variables were given as median (interquartile range, IQR quartile 25-quartile 75) and analyses were made with Fisher’s exact test. Categorical variables were given as numbers and percentages and analyzed with Mann Whitney’s test.

The most common intravenous antibiotic used was cefazolin (12 patients, 60%), followed by amoxicillin (3 patients, 15%). A switch to oral therapy was possible for all patients. Levofloxacin with clindamycin was the main oral combination prescribed (4 patients, 44%), followed by levofloxacin with rifampin (3 patients, 33%).

Eleven patients (55%) were treated with single antibiotic therapy, with clindamycin being the main antibiotic used (5 patients, 46%) (Table 2).

**Table 2:** Treatment and outcomes

	Overall (*N* = 20)	Single therapy (*N* = 11)	Combination therapy (*N* = 9)	*P* value
Duration of intravenous antibiotics, days (median [IQR])	6.00 [4.75;7.25]	7.00 [4.50;12.5]	5.00 [5.00;6.00]	0.25
Duration of oral antibiotics, days (median [IQR])	34.0 [29.5;36.0]	33.0 [27.0;35.5]	36.0 [33.0;37.0]	0.12
Total duration on antibiotics, days (median [IQR])	40.0 [38.0;41.5]	40.0 [38.0;41.0]	41.0 [38.0;43.0]	0.76
**Intravenous antibiotics used**				
Cefazoline (n, %)	12 (60.0%)	6 (54.5%)	6 (66.7%)	0.24
Amoxicillin (n, %)	3 (15.0%)	3 (27.3%)	0 (0.00%)	
Cloxacillin (n, %)	2 (10.0%)	0	2 (22.2%)	
Vancomycin (n, %)	2 (10%)	1 (9%)	1 (11.1%)	
Daptomycin (n, %)	1 (5.0%)	1 (9%)	0	
Switch to oral antibiotics (n, %)	20 (100%)	11 (100%)	9 (100%)	1.00
**Oral antibiotics used**				
Levofloxacin (n, %)	11 (55.0%)	3 (27.3%)	8 (88.9%)	0.08
Clindamycin (n, %)	9 (45.0%)	5 (45.5%)	4 (44.4%)	
Amoxicillin (n, %)	3 (15.0%)	2 (18.2%)	1 (11.1%)	
Rifampin (n, %)	3 (15.0%)	0	3 (33.3%)	
Doxycycline (n, %)	1 (5.0%)	0	1 (11.1%)	
Linezolid (n, %)	1 (5.0%)	1 (9.09%)	0	
Secondary adverse events imputed to antibiotics	1 (5.00%)	1 (11.1%)	0 (0%)	
Patients undergoing new surgical debridement (n, %)	4 (20.0%)	3 (27.3%)	1 (11.1%)	0.59
Delay between initial revision and new surgical debridement (days) (median [IQR])	20 [10.5 ; 114.8]	12 [9 ; 20]	376	0.5
Patients with microbiological failure (n, %)	0	0	0	
Deaths from any cause at 1 year (n, %)	1 (5.0%)	0	1 (11.1%)	
Primary endpoint (n, %)	1 (5.0%)	0	1 (11.1%)	
Deaths from any cause during follow-up (n, %)	2 (10.0%)	0	2 (22.2%)	
Duration of follow-up, days (median [IQR])	1024 [580;1512]	862 [573;1157]	1493 [699;1715]	0.18

Regular doses of oral antimicrobial therapy are listed here: levofloxacin 750 mg/d, clindamycin 600 mg 4 times a day, amoxicillin 100–150 mg/kg/d, rifampicin 600 to 900 mg/d, doxycycline 200 mg/d, linezolid 600 mg 2 times a day.

We reported only one severe adverse event imputed to levofloxacin in the single antibiotic group (sudden cardiac arrest) requiring a switch to clindamycin.

Only one patient reached the primary endpoint (death at 135 days) due to end-stage heart failure without any link to spinal infection (Table 2). Four patients (20%) underwent new surgical debridement (3/4 in the single antibiotic group) because of surgical failure, with no micro-organisms found on surgical cultures. We didn’t report any proved persistent infection or relapse during follow-up on both treatment groups. Two out of 3 patients needing new surgical intervention in the single antibiotic group were still treated by intravenous antibiotic treatment at the time of the surgery. Therefore, these new interventions could not be attributable to single oral antimicrobial therapy. We reported 1 death (10% total mortality rate) after one year of follow-up, due to complications of underlying diseases. There was no outcome difference between single antibiotic therapy and combination therapy (Table 2).

## Discussion

We here described the results of a retrospective cohort of staphylococcal implant-free pSSI treated with either single or combination antibiotics. Patients had few underlying diseases and good outcome with a 95% clinical cure rate after one year. We described four revision surgeries, none proved due to persistent infection or relapse. Despite a long median length of follow-up of almost 3 years, only 1 death occurred during the first year, unrelated to surgery or antibiotic treatment.

Overall, there was a low number of failures reported in contrast to previous studies with failure rates ranging from 5 to 85% in case of retained instrumentation [3, 8–10], explained by several factors: (i) we may have missed long-term relapses, (ii) patients were not at high risk of relapse due to low number of underlying diseases and absence of methicillin-resistant S. *aureus* [3, 11], (iii) there was no retention of spinal implants in our study [9].

To our knowledge, this is the first study focusing on antibiotic treatment modalities of implant-free post-surgical spinal infections. One of the limitations of our study is that rifampin was not systematically used in antibiotic combinations, with clindamycin often used as a second therapeutic agent. However clindamycin has been used for years in osteoarticular infections with high cure rate [12]. It is known to have excellent bone penetration, oral bioavailability and few side effects [12, 13]. Moreover, although rifampin’s antibiofilm action is recommended to treat spinal infection with retention of foreign implants, there is no clear data regarding the benefit of rifampin in implant-free spinal infections. Indeed, while combination treatment with biofilm-active antibiotics was associated with lower treatment failure in some studies combining post-surgical infections with and without implant removal [11, 14], this effect was not significant in case of methicillin-susceptible *S. aureus* infection or implant removal in the study by Cho et al. [11].

In our study, clindamycin and levofloxacin were the main single antibiotics prescribed, allowing conclusion of efficacy to be drawn on these two antibiotics only. One quarter of *S. aureus* isolates were susceptible to penicillin G and interestingly 3 out of them were treated with amoxicillin with satisfactory outcome.

Although we presented a small sample size, with various staphylococci identification and with various antimicrobial use, it is however to date the largest study of implant-free staphylococcal pSSI treated with a single antibiotic therapy. We suggest that a single antibiotic treatment with clindamycin or levofloxacin could be as efficient as a combination therapy in implant-free post-surgical staphylococcal spinal infection in patients with few underlying diseases. Combination therapy could lead to more iatrogenic complications, alterations of gut microbiota and higher costs. A randomized control trial comparing these antibiotics to a rifampin-containing combination should be conducted to confirm these results.

### Electronic supplementary material

Below is the link to the electronic supplementary material.

Flow chart

## Data Availability

The datasets used and analysed during the current study are available from the corresponding author on reasonable request.

## Abbreviations

pSSI: Post-surgical spinal infections
