# Supplementary figures and images for: Efficacy of single antibiotic therapy versus antibiotic combination in implant-free staphylococcal post-surgical spinal infections: a retrospective observational study
Source: BMC Infect Dis. 2024 Jan 8;24:62. doi: 10.1186/s12879-024-08977-y (PMC10775553; doi:10.1186/s12879-024-08977-y)

**Supplementary Figure 1**


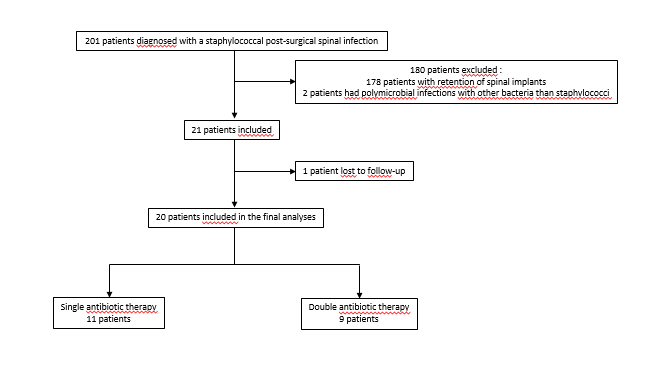


*Flow chart of the study*

Supplement: Supplementary file 1 — Flow chart [file 12879_2024_8977_MOESM1_ESM.docx]
